# A novel interactive educational system in the operating room–the IE system

**DOI:** 10.1186/s12909-016-0561-0

**Published:** 2016-02-02

**Authors:** Takayuki Nakayama, Noboru Numao, Soichiro Yoshida, Junichiro Ishioka, Yoh Matsuoka, Kazutaka Saito, Yasuhisa Fujii, Kazunori Kihara

**Affiliations:** Department of Urology, Tokyo Medical and Dental Graduate School, 1-5-45 Yushima, Bunkyo-ku, Tokyo 113-8519 Japan

**Keywords:** Surgical education, Medical students, Surgery

## Abstract

**Background:**

The shortage of surgeon is one of the serious problems in Japan. To solve the problem, various efforts have been undertaken to improve surgical education and training. However, appropriate teaching methods in the operating room have not been well established. The aim of this study is to assess the utility of a novel interactive educational (IE) system for surgical education on urologic surgeries in the operating room.

**Methods:**

A total of 20 Japanese medical students were educated on urologic surgery using the IE system in the operating room. The IE system consists of two parts. The first is three-dimensional (3D) magnified vision of the operative field using a 3D head-mounted display and a 3D endoscope. The second is interactive educative communication between medical students and surgeons using a small-sized wireless communication device. The satisfaction level with the IE system and the physical burden on medical students was examined via questionnaire.

**Results:**

All students utilized the IE system in urologic surgery and responded to the survey. Most students were satisfied with the IE system. They also felt more welcomed by the surgeon when using the IE system than when not using it. No major unpleasant symptoms were observed but five students (25 %) experienced mild eye fatigue as a result of viewing the medical images.

**Conclusions:**

The IE system has the potential to motivate students to become interested in surgery and could be an efficient method of surgical education in the operating room.

## Background

In recent years, several countries have reported shortages of surgeons [1, 2]. In Japan, Mizuno et al. reported that between 1996 and 2006, the number of surgeons fell by 14.8 %, from 51,101 to 43,528 [3]. The causes for the shortage of surgeons in Japan are presumed to be long hours, excessive emergency surgeries, and high risk of a lawsuit [4]. Furthermore, Ide et al. reported that although more women enrolled in the specialty of surgery, a lower number of men chose it [5]. Ide et al. also showed that female surgeons tended to change their area of specialty (such as to primary care) or retire. Thus, the shortage of surgeons would continue and remain a matter of significant concern for surgical care at present and in the future. To resolve this serious problem, it is important to improve surgical education and to attract medical students to undergraduate surgical training.

One of the most important factors in medical training success is student motivation. Despite this, Chapman et al. reported that, of 267 medical students who received surgical education in operating rooms in the United Kingdom, 115 (43.1 %) had bad or unfavorable experiences [6]. Common themes included feeling intimidated, unwelcome, or ignored by staff, unrealistic expectations of knowledge, and poor or inadequate learning experiences. It is apparent that this insufficient surgical education leads to a decrease in the number of residents who desire to be a surgeon. Therefore, to increase the number of surgeons, sufficient and favorable surgical education should be created.

Medical treatment has become increasingly high-tech. Various high-tech equipment has a great potential to advance surgical education as well as surgery itself. To provide sufficient surgical education, visual information on the operation and interactive communication between the trainee and participants in the operation is important. Recently, modern head-mounted displays (HMDs) that provide high-resolution 3D images have become commercially available at an affordable cost. We previously demonstrated the clinical application of the latest HMDs for medical providers in urologic surgeries [7–9]. The significant utility of the HMD motivated us to use it for surgical education. For interactive communication, we believe that wireless and wearable communication devices are beneficial. Thus, we examined the initial experience of surgical education using a combination of 3D HMDs and wireless and wearable communication devices.

## Methods

This study was approved by the Ethics Committee of Tokyo Medical and Dental University. The participants included a total of 20 fifth-year or sixth-year medical students (3 women and 17men) from the medical school of Tokyo Medical and Dental University, Tokyo. All patients previously experienced conventional surgical education without HMDs. Each student voluntarily took part in this study and provided informed written consent with regard to the project.

**Fig. 1:**
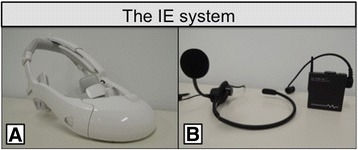
The interactive educational system (IE system): **a**. Head-mounted display. **b**. G-TALK

During endoscopic urologic surgery, including endoscopic nephrectomy and endoscopic radical prostatectomy, surgeons wore a HMD, as usual. As a main monitor, we have used HMDs instead of standard surgical monitors in endoscopic urologic surgery since 2012. Medical students also wore HMDs in the current study. We used three types of HMDs, including HMZ-T1, HMZ-T2, and HMM-3000MT (Sony Corporation, Tokyo, Japan) (Fig. 1a). HMZ-T1 or HMZ-T2 is a 3D-HMD for consumer use and its weight is 420 g or 330 g, respectively. HMM-3000MT is an HMD for medical use with a weight of 490 g. These three HMDs are binocular HMDs composed of a 0.7-inch organic light-emitting diode screen (resolution: 1,280 × 720 pixels) for each eye, which provides the wearer high-contrast and sharp images in front of the eyes. In the current analysis, we used two types of high-definition 3D endoscopes. The first is a rigid 3D endoscope (Shinko Optical, Tokyo, Japan), and the second is a flexible 3D endoscope (Olympus, Tokyo, Japan). Surgeons and medical students can view the same 3D surgical view by a combination of the 3D-HMDs and the 3D endoscope.

As a wireless and wearable communication device, we selected a G-TALK (KOHNAN Electronic Corporation, Japan) (Fig. 1b). The G-TALK is a compact-sized 2.4 GHz digital transceiver that provides clear sound quality with a weight of 70 g. Its height, width, and depth are 65, 59, and 18 mm, respectively. A dedicated headset with a microphone can be attached to the G-TALK. Medical students and one of the operators (the surgical educator) place the G-TALK in a pocket and wear a headset with a microphone attached to it. Three surgical educators (T.N., N.N. and K.S) were enrolled in this study. The participants were randomly assigned to the surgical team including the surgical educators. Interactive conversation is possible using the G-TALK. Using a combination of the 3D-HMDs with a 3D endoscope and the G-TALK, we constructed an interactive educational (IE) system.

During operations, medical students were surgically educated using the IE system. Medical students can see a magnified 3D view while they engage in interactive communication with the surgical educator, who is one of the participants in the operation. After the operation, medical students completed a questionnaire that evaluated (1) satisfaction of medical training without the IE system (previous experience) (Question A-C), (2) satisfaction of medical training with the IE system (Question D-J), and (3) unpleasant symptoms (Question a-c) (Table 2). A paired- *t* test was performed to assess the statistical significance by using JMP Statistical Software Release 10. (SAS Institute, Inc., Cary, NC). Significance was defined as a *p* value less than 0.05.

## Results

Table 1 shows student characteristics and types of surgery in the current study. All students utilized the IE system during an operation and completed the questionnaire. The results of the answers are shown in Table 2. The results of questions A reveal that many students hesitated to ask questions of the surgeon during surgery (Question A: average point 2.6) and did not feel welcome by the surgeon during previous surgical training without the IE system (Question C: average point 1.6).

**Table 1:** Participants’ characteristicsᅟ

Gender (n) (%)	
Male	17 (85)
Female	3 (15)
School year (n) (%)	
5	12 (60)
6	8 (40)
Surgery type (n) (%)	
Partial nephrectomy	12 (60)
Radical nephrectomy	6 (30)
Radical prostatectomy	2 (10)
Average Used time of the IE system (min)	103 (5–420)

*IE system* Interactive educational system

**Table 2:** Questionnaire given to the students and questionnaire data analysis resultsᅟ

	Qustions		Average score
Questions for medical training without IE system (previous experience)	A. Have you experienced hesitation to raise a question with surgeon during a surgery?		2.6
	1. Much hesitation, 2. Occasional hesitation, 3. A little hesitation, 4. Little hesitation, 5. No hesitation		
	B. Did you have confidence in anatomical knowledge?		2.0
	1. No confidence, 2. Little confidence, 3. A little confidence, 4. Some confidence, 5. Much confidence		
	C. Have you felt unwelcome by surgeon?		1.6
	1. Unwelcome, 2. A little unwelcome, 3. No feeling, 4. A little welcome, 5. Welcome		
Questions for the current training with IE system	D. Did you undergo medical training in the operating room with interest?		4.5
	1. Very boring, 2. A little boring, 3. No feeling, 4. A little interesting, 5. Very interesting		
	E. Could current training improve your anatomical knowledge?		4.3
	1. Not at all, 2. Little, 3. A little, 4. Some, 5. Very		
	F. Was the IE system easy to use?		3.6
	1. Very difficult, 2. Difficult, 3. A little difficult, 4. Easy, 5. Very easy		
	G. Did the IE system give you the motivation?		4.4
	1. Not at all, 2. Little, 3. A little, 4. Some, 5. Very		
	H. Did you have hesitation to raise a question with surgeon during a surgery?		3.6
	1. Much hesitation, 2. Occasional hesitation, 3. A little hesitation, 4. Little hesitation, 5. No hesitation		
	I. Did you felt unwelcome by surgeon?		3.4
	1. Unwelcome, 2. A little unwelcome, 3. No feeling, 4. A little welcome, 5. Welcome		
	J. Do you want to use the IE system in the future?		3.9
	1. Not at all, 2. Little, 3. A little bit, 4. Some, 5. Very much		
Questions for adverse events from using HMD		No. of answer “Yes” (%)	
	a. Did you feel the feeling of discomfort by using the HMD		2 (10)
	Yes or No		
	b. Did you feel the fatigue of your body by using the HMD		3 (15)
	Yes or No		
	c. Did you feel the fatigue of your eye by using the HMD		5 (25)
	Yes or No		

Paired *t*-test: Question A vs H; *p* =0.0042

Question B vs E; *p* <0.0001

Question C vs I; *p* <0.0001

*IE system* Interactive educational system

*HMD* head-mounted display

In contrast, the results of questions D to J revealed that the surgical training with the IE system made students interested (Question D: average point 4.5) and motivated (Question G: average point 4.4). They also felt more welcome by the surgeon when using the IE system (Question I: average point 3.4) than when not using it (Question C: average point 1.6) (*p* <0.0001). In addition, using the IE system, they felt less hesitant to ask a question (Question H: average point 3.6) than when not using it (Question A: average point 2.6) (*p* =0.0042). The IE system improved perception of the anatomical knowledge (Question E: average point 4.3) of the students who had little confidence in their knowledge more than when not using it (Question B: average point 2.0) (*p* <0.0001).

Ten percent of students felt discomfort wearing the 3D-HMD. Fifteen and twenty-five percent of students felt fatigue in their body and eyes, respectively (Table 2). The incidence of adverse event was not correlated with gender (Question a: *p* =0.4068, b: *p* =0.3022, c: *p* =0.7246). Used time of the IE system was associated with the feeling of discomfort (Question a: *p* =0.0368), but not with the fatigue of their body (Question b: *p* = 0.0831) and eye (Question c: *p* =0.2301).

## Discussion

In the current study, it has been demonstrated that the IE system can have a positive effect in terms of student interest and educational success in surgical training. Many students also felt unwelcomed by surgeons at previous surgical training. While using the IE system that enables interactive communication, good communication between surgeons and medical students can be built. We believe that this can improve the satisfaction of medical students receiving surgical education.

A combination of a 3D-HMD and a 3D endoscope can offer magnified 3D high-resolution images. Several reports have shown that 3D representations are increasingly used not only in the clinical setting but also in student education [10–14]. The benefits of 3D learning tools have been constantly demonstrated through well-powered randomized studies [10, 15–18]. Blavier et al. showed the surgical education by using 3D images improve the feeling of mastery, familiarity, satisfaction, self-confidence and facility for medical student, which are essential factors of well-being and motivation [18]. In the current study, the IE system also improved motivation as well as anatomical knowledge for surgery. One of the reasons is thought to be the 3D images offered by the IE system. To enhance student’s motivation, we consider the use of 3D images instead of 2D images is encouraged in surgical education if possible. The positive feeling for medical student may help in solving the problem of shortage of surgeon.

Several studies have recently reported the potential use of see-through HMD, which is worn like conventional glasses, in surgery [19, 20]. Although see-through HMD has the advantage of lightness, it has the disadvantages of lower pixel counts than the HMDs we used in the current study and of two-dimensional display. It has been shown that 3D presentations enhance the understanding of complex anatomical structures [10, 21]. In this context, we believe that lightweight and high-resolution 3D-HMDs could be promising tools for surgical education.

A surgeon is under a great deal of strain while performing surgery and is not able to engage in discussion during surgery. Therefore, it is natural for medical students to be hesitant to ask surgeons questions. In the current study, G-TALK contributed to smooth discussions between medical students and surgeons because it enables telecommunication with high sound quality.

Our report has several limitations. Foremost, the limitations of the small sample size should be considered. A larger study cohort is needed to confirm our results. Second, a problem common to all studies that investigate perceptions and attitudes is the role of personal bias. For example, an individual’s interest in surgery may be biased. Studies that examine student interest are worth doing. Third, the data gathered through a questionnaire include recall bias. Moreover, it is a potential bias that our data were collected from two different situations including the previous surgical experience and the current study. Therefore, our results should be confirmed by the without these biases. Fourth, our study focused on the technology using surgical education, however, how the technology was used should be further established.

## Conclusions

The IE system using a 3D-HMD with a 3D endoscope and a small-sized wireless communication device can offer a magnified 3D view of the operative field and interactive communication between surgeons and medical students. This system made medical students motivated and therefore has the potential to improve surgical education in the operating room.

## Abbreviations

HMD: head-mounted display
IE system: novel interactive educational system
